# Effects of Dietary Supplementation with *Agaricus sylvaticus* Schaeffer on Glycemia and Cholesterol after Streptozotocin-Induced Diabetes in Rats

**DOI:** 10.1155/2014/107629

**Published:** 2014-05-26

**Authors:** Marcelo Betti Mascaro, Cristiane Miranda França, Kamilla F. Esquerdo, Marx A. N. Lara, Nilsa S. Y. Wadt, Erna E. Bach

**Affiliations:** ^1^Universidade Nove de Julho (UNINOVE), 01504-001 São Paulo, SP, Brazil; ^2^Postgraduate Program in Biophotonics Applied to Health Sciences, Universidade Nove de Julho (UNINOVE), 01504-001 São Paulo, SP, Brazil

## Abstract

This study evaluated the effect of the *Agaricus sylvaticus* (sun mushroom) on biochemical tests of the plasma and on the morphology of the pancreas in an experimental model of type I diabetes mellitus (DM1) induced by streptozotocin. One gram of dry *A. sylvaticus* was homogenized and mixed with the chow. Male Wistar rats were allocated as follows: normoglycemic control that received commercial chow; normoglycemic control group that received chow with *A. sylvaticus;* diabetic group that received commercial chow; and diabetic group that received chow with *A. sylvaticus*. Weight, food, and water consumption were measured every two days. Blood glucose levels were measured twice a week. After 30 days, the animals were euthanized and blood was collected for the analysis of cholesterol, HDL, triglycerides, blood sugar, glutamic-pyruvic transaminase (GPT), alkaline phosphatase, iron, transferrin, and urea. The pancreas was processed for microscopic analysis. *A. sylvaticus* modulated the levels of cholesterol, HDL, triglycerides, blood sugar, GPT, alkaline phosphatase, iron, transferrin, and urea to levels similar to those found in the controls and led to compensatory hyperplasia of the islets of Langerhans. *A. sylvaticus* is potentially beneficial in the control of type 1 diabetes, and it may also prevent pancreas damage.

## 1. Introduction

Diabetes mellitus is a group of metabolic diseases characterized by hyperglycemia resulting from deficient insulin secretion and/or action [1]. This disease is associated with a reduction in quality of life and an increase in risk factors for comorbidities and mortality. Long-term hyperglycemia is an important factor to the development and progression of microvascular and macrovascular complications [2, 3].

A number of natural products have been employed for the prevention and treatment of diseases. According to the World Health Organization (WHO), nearly 80% of the population worldwide relies on medicinal herbs as their primary form of health care [4]. Among such products, mushrooms, especially basidiomycetous fungi, have attracted attention due to their low toxicity and high degree of specificity in the activation of the human immune system [4].

The basidiomycete* Agaricus blazei* Murrill is currently cultivated in different regions of Brazil [5]. Similar mushrooms as* Agaricus sylvaticus* (Schaeffer) and* Agaricus brasiliensis* [6] are equally distributed on the country. These three species of mushrooms are popularly denominated “sun mushrooms” [5, 7, 8].

The qualitative characteristics of mushrooms are influenced by the species, strain, postharvest processing, age, environment, and locality [7]. Oliveira et al. (1999) [9] analyzed the nutritional value of* A. blazei* and concluded that this mushroom is rich in proteins (30%), fibers (14.6%), and minerals (P = 2.34%; Ca = 0.07%; S = 0.29%; Zn = 86.9 mcg; Fe = 79.13 mcg) and has a low content of lipids (1.48%). Subsequent studies report different figures, but all agree that the sun mushroom has a rich chemical composition with a wide variety and quantity of minerals and protein [10–12].

*Agaricus blazei* is rich in immunomodulating polysaccharides and *β*-glucans and has been shown to have antitumor, anti-infection, antiallergic, and antiasthmatic properties in mouse models in addition to anti-inflammatory effects in individuals with inflammatory bowel disease [13, 14]. *β*-Glucan extracts from* A. blazei* have been reported to reduce blood glucose, triglycerides, and cholesterol levels and exhibit insulin-like action. To clarify the antidiabetic efficacy and hypoglycemic mechanisms, Niwa et al. (2011) [14] submitted rats with streptozotocin-induced diabetes to daily oral feeding with powdered sweet potato (*Ipomoea batatas*) and* A. blazei* and found a decrease in the fasting plasma glucose and glycated hemoglobin as well as the restoration of body weight loss due to diabetes. The authors concluded that the hypoglycemic effects of these products result from the suppression of oxidative stress and proinflammatory cytokine production, followed by an improvement in pancreatic *β*-cell mass.

The aim of the present study was to evaluate the effect of* Agaricus sylvaticus* on the blood sugar, cholesterol, HDL, triglycerides, glutamic-pyruvic transaminase (GPT), alkaline phosphatase, iron, transferrin, and urea level in the plasma of rats with type 1 diabetes mellitus (DM1) induced by streptozotocin. Moreover, a morphological analysis of the pancreas of the animals was made.

## 2. Materials and Methods

### 2.1. Preparation of* Agaricus sylvaticus* and Analysis

*A. sylvaticus* was cultivated in the state of Rio Grande do Sul (Brazil). After harvesting, the fruiting bodies were washed in water, dried, and finely milled in a hammer mill equipped with a 1 mm mesh stainless steel sieve (Vitali Cogumelos, Rio Grande do Sul, Brazil). The sifted powder was stored dry until further use. Dry weight was normally between 7 and 10% of fresh weight. One gram of dry mushroom was homogenized in 40 mL of water, maintained at 4°C for one hour, and filtered with Whatman number 1 filter paper.

The extract was read in a spectrophotometer UV/light 270–580 nm (Beckman DU 530, Beckmann Instruments Inc.) and 1 mL was submitted to Sephadex G-50 chromatography gel filtration (2.0 × 10 cm) at 25°C. Elution was performed with phosphate buffer (10 mmol/L, pH 7). Flow was 2 mL for 1 minute and samples were collected in 20 tubes. Pooled fractions were submitted to the analysis of proteins [15] and phenols [16]. Lever's method was used for beta-glucan [17], which involved 0.1 mL of extract diluted in 50 mM sodium acetate, pH 5, mixed with 0.1 mL of beta-glucanase enzyme (Sigma), and kept for 30 min at 37°C. At the end of the reaction, 1.5 mL of p-hydroxybenzoic acid hydrazide (Sigma) was added. The samples were heated to 100°C for 10 min and cooled to room temperature and absorbance was read at 410 nm. Optical density was converted into mg of reducing sugar using a standard curve prepared with glucose and laminarin (Sigma). One glucanase unit is defined as the amount of enzyme that produces 1 mg/mL from laminarin glucose. Insoluble beta-glucan was calculated in a sample without enzyme.

Thin-layer chromatography was performed on Merck silica gel 60 F254 plates (10 cm × 10 cm). Aliquots of standard p-coumaric acid, kaempferol, chlorogenic acid, salicylic acid, rutin and the extracts were applied as spots at the origin on a plate and developed with butanol-acetic acid-water (BAW 4:1:5) in a presaturated chromatographic chamber. Developed plates were dried with a stream of hot air (hair dryer) and viewed with ultraviolet (UV) light and ferric chlorite (1% in alcohol). The Biorad software program was used to determine the area of each spot on the plate. Each *R*_*f*_ value was compared to a curve prepared using the standard p-coumaric acid (*R*_*f*_ = 0.88), kaempferol (*R*_*f*_ = 0.94), chlorogenic acid (*R*_*f*_ = 0.52), salicylic acid (*R*_*f*_ = 0.77), and rutin (*R*_*f*_ = 0.48) using the standard (10 *μ*L of each solution) p-coumaric acid. The extract was also quantified in a UV/light 275–600 nm spectrophotometer (Beckman DU 530-Beckmann Instruments Inc.), with the readings compared to the peak absorbance of the standards.

Based on the results of the biochemical analysis, 1 g of mushroom powder was extracted with 40 mL of cool water and mixed with 9 g of previously crushed, sieved commercial feed, forming round pieces, and placed in a Pyrex container. All samples were dried in an oven at 60°C. The chow was then weighed and placed in the animal cages. One gram of feed with* A. sylvaticus* was diluted in 2 mL of water and the quantity of beta-glucan was then analyzed.

One gram of the commercial chow (Bio Base, São Paulo, SP, Brazil) contained protein (22 mg), calcium (1 mg), phosphorus (0.8% mg), fiber (0.8 mg from rice bran, wheat, and corn), minerals (1 mg), and 12% moisture.

### 2.2. Animals

Male Wistar rats aged four weeks and weighing 250 to 280 g were obtained from the University Nove de Junho (UNINOVE) animal lodging facility. The animals were kept in polypropylene cages (two to three animals per cage) covered with metallic grids in a room maintained at 23°C, 55 ± 10% relative humidity, and a 12 h light/dark cycle. The animals had free access to food and water for two weeks before beginning the study. The UNINOVE Ethics Committee for Animal Research approved the protocols used in this study (process numbers: 34/2010 and 20/2012).

### 2.3. Diabetes Type 1 Induction and Experimental Groups

The animals were divided into four groups of five rats. In two groups, the animals were fasted for 12 hours and chemical diabetes was induced through an intraperitoneal injection of streptozotocin (50 mg/kg) (STZ, Sigma). The STZ solution was prepared immediately prior to injection by dissolving the drug in a fresh, cold citrate buffer, pH 4.5. After 72 hours, blood glucose levels were measured using a portable glucose meter (One Touch II; Johnson & Johnson, Milpitas, CA). For such, the distal part of the tail was gently snipped; the first blood drop was discarded and the second was absorbed by a test strip inserted in the glucose meter. Rats were considered diabetic when the blood glucose level was at least 250 mg/dL.

The four groups were composed as follows: (1) C: normoglycemic control that received unaltered commercial chow; (2) CA: normoglycemic control group that received commercial chow with* A. sylvaticus;* (3) D: diabetic group that received unaltered commercial chow; and (4) DA: diabetic group that received commercial chow with* A. sylvaticus*. The groups were evaluated for 30 days. Food and water consumption were measured every two days. Blood glucose levels and weight were measured twice a week, always at 11:00 a.m.

### 2.4. Euthanasia and Sample Collection

At the end of the experimental period, the animals were anesthetized with a lethal dose of a cocktail containing ketamine (1 mg), xylazine (5 mg), and acepromazine (0.2 mg). Thoracotomy was performed. Blood was collected from the left ventricle and centrifuged. The plasma was removed and stored at −20°C for no longer than three days before the assay. Total cholesterol, triglycerides, HDL cholesterol, urea, creatinine, iron, and transferrin were measured using test kits (Labtest Diagnostica).

### 2.5. Histological Analysis

After euthanasia, the pancreas was removed, rinsed with water, fixed in a 10% buffered formalin solution, and embedded in paraffin wax. Sections measuring 5 *μ*m in thickness were prepared and stained with hematoxylin and eosin [18].

The three best sections from each animal were submitted to microscopic examination. Five nonconsecutive areas of each sample were photographed (Leica Microsystems, Wetzlar, Germany) (160x magnification) and analyzed using the Image J program with the “measure” plug-in (version 1.45q, free software, NIH, Bethesda, Maryland, USA) for the measurement of the islets of Langerhans. The number of islets and nuclei in each islet was also counted.

### 2.6. Statistical Analysis

Statistical analysis was performed using the Assistat-2012 software and involved ANOVA, the Student's *t*-test, and Tukey's test. Statistical significance was determined by *P* values <0.05 and <0.01.

## 3. Results

### 3.1. Analysis of* A. sylvaticus*

The fruiting bodies had a light yellow color, indicating that drying was carried out at 40 to 60°C to avoid caramelizing the product. The finely milled powder demonstrated solubility in water and was therefore used in the present study.

For the biochemical analysis, the extract from* A. sylvaticus* was made with cool water to extract the polysaccharide bound to protein without the action of phenols or peroxidase activity, thereby obtaining an extract with a light yellow color. When hot water was used, more phenols were extracted and the protein was decreased, giving a brown coloration to the solution corresponding to the oxidation of molecules [8].

**Figure 1:**
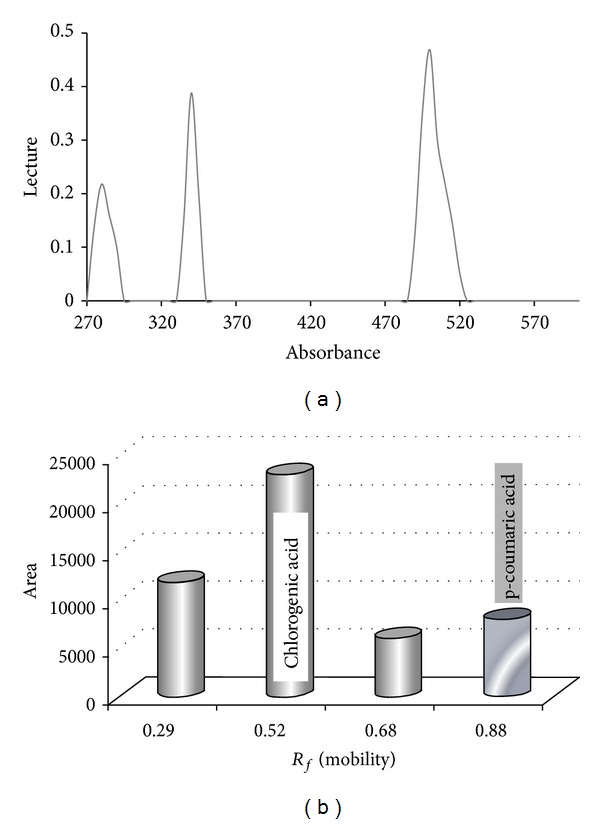
(a) Spectrophotometer readings of extract of* A. sylvaticus* at 270 to 600 nm; (b) thin-layer chromatography analysis of 10 *μ*L of extract: *R*_*f*_ (mobility) and area (mm^2^); BAW reagent (4:1:5 organic phase), standard chlorogenic acid (*R*_*f*_ = 0.48), and p-coumaric acid (*R*_*f*_ = 0.88) absorbed at 340 nm.

The extract was first submitted to UV/light spectrophotometry at wavelengths of 275 to 600 nm, demonstrating three peaks: (1) a peak at A280 nm correlated with proteins; (2) a peak at A340 nm correlated with chlorogenic acid and p-coumaric acid; (3) peaks at A500 nm that were not identified. Phenols were confirmed by thin-layer chromatography using BAW (4:1:5) and the results demonstrated four compounds: *R*_*f*_ = 0.29 (unknown identity), *R*_*f*_ = 0.52 (correlated with the chlorogenic acid standard), *R*_*f*_ = 0.68 (unknown identity), and *R*_*f*_ = 0.88 (correlated with p-coumaric acid) (Figure 1). The biochemical analysis of the mushroom powder revealed 1.15 mg of proteins, 0.96 mg of phenols, 224.03 mg of beta-glucan, 31 mg of fiber, p-coumaric acid, chlorogenic acid, and two unknown compounds.

**Figure 2:**
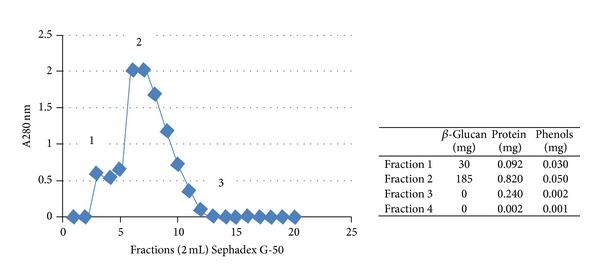
Graph: Sephadex G-50 chromatography column and pooled fraction 1 (tubes 3 and 4), fraction 2 (tubes 6 to 8), fraction 3 (tubes 10 to 12), and fraction four (tubes 13 to 16). Table: mg of beta-glucan, protein, and phenols in each fraction.

One mL of crude extract was submitted to chromatography gel filtration and separated into four pooled fractions: (1) external volume (fraction tubes 3 and 4), (2) internal volume (tubes 6, 7, and 8), (3) tubes 10, 11, and 12, and (4) tubes 13, 14, 15, and 16. All fractions were submitted to analyses for the quantification of protein, phenols, and beta-glucan (Figure 2).

### 3.2. Animal Weight, Food, and Water Consumption

**Figure 3:**
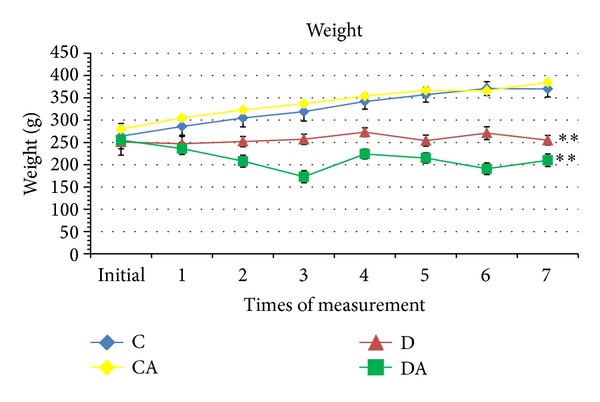
Body weight of rats submitted to treatments (C: normoglycemic control group having received unaltered commercial chow; CA: normoglycemic control group having received chow with* A. sylvaticus*; D: diabetic group having received unaltered commercial chow; DA: diabetic group having received commercial chow with* A. sylvaticus*). Measurements were taken twice a week for four weeks. Weight values are mean of 5 animals. The first measurement is prior to the introduction of* Agaricus* in the chow but when the diabetic induction had already been done. “∗∗” on lines indicates statistically significant differences among all groups (*P* < 0.05; ANOVA + Tukey's test).

**Figure 4:**
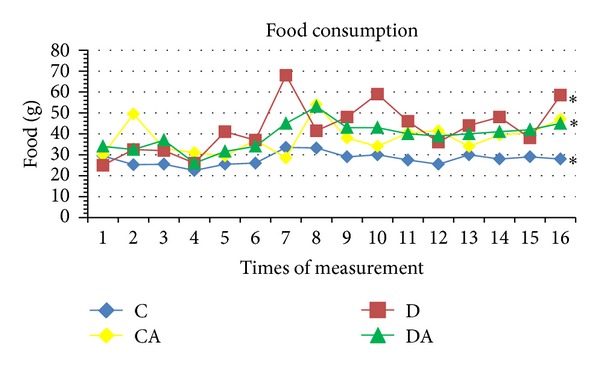
Food consumption of rats submitted to treatments (C: normoglycemic control group having received unaltered commercial chow; CA: normoglycemic control group having received chow with* A. sylvaticus*; D: diabetic group having received unaltered commercial chow; DA: diabetic group having received commercial chow with* A. sylvaticus*). Measurements were taken every two days for 4 weeks. Values are mean of 5 animals. “∗” on lines indicates statistically significant differences between the group and the control (*P* < 0.05; ANOVA + Tukey's test).

**Figure 5:**
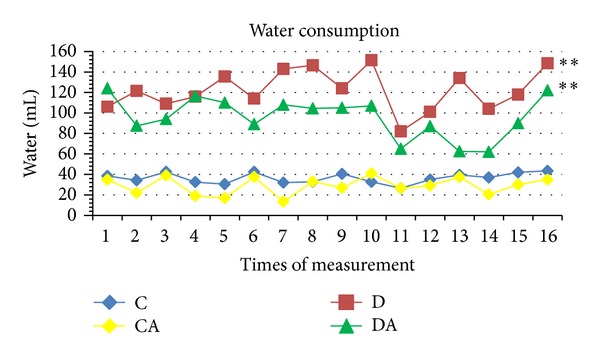
Water consumption of rats submitted to treatments (C: normoglycemic control group having received unaltered commercial chow; CA: normoglycemic control group having received chow with* A. sylvaticus*; D: diabetic group having received unaltered commercial chow; DA: diabetic group having received commercial chow with* A. sylvaticus*). Measurements were taken every two days for 4 weeks. Values (mL) are mean of 5 animals. “∗∗”on lines indicates statistically significant differences among all groups (*P* < 0.05; ANOVA + Tukey's test).

In the control groups (C and CA), weight increased throughout the experimental period, with no significant differences between the two groups. Significant differences were found in the comparison of C with the D and DA groups (*P* < 0.05). Weight in the D group remained largely stable throughout the period, whereas a slight decrease was found in the DA group. Moreover, a significant difference was found between the D and DA groups (Figure 3). Interestingly, while the animals in the C and CA groups had similar increases in weight, the CA group consumed a significantly greater amount of food than the C group. As expected, food and water consumption were greater in the D group. However, when the diabetic rats began to consume* A. sylvaticus* (DA group), food and water intake decreased (Figures 4 and 5).

### 3.3. Biochemical Analysis of Plasma

Table 1 displays the results of the biochemical tests at the time of euthanasia.

#### 3.3.1. Kidney Function Markers: Creatinine and Urea

A significant increase was found in serum urea between the D group and the other groups. Moreover, serum urea was significantly lower in the DA group when compared to the D group (*P* < 0.01). In contrast, no significant differences among groups were found regarding serum creatinine (Table 1).

#### 3.3.2. Markers of Tissue Oxygenation: Transferrin and Iron

Significant reductions were found in serum transferrin and iron between the D group and the other groups, whereas no significant differences were found among the C, CA, and DA groups (Table 1).

#### 3.3.3. Markers of Liver Function: Alkaline Phosphatase and Glutamic-Pyruvic Transaminase (GPT)

The alkaline phosphatase level in the D group was strongly elevated in comparison to the other groups, whereas no significant differences were found among the C, CA, and DA groups. The same pattern was found with regard to GPT (Table 1).

#### 3.3.4. Total Cholesterol, HDL Cholesterol, and Triglycerides

Significant increases in serum cholesterol and triglycerides and a significant reduction in HDL were found in the D group in comparison to the other groups. The DA group exhibited a reduction in triglycerides and total cholesterol as well as an increase in HDL. A slight decrease in cholesterol and increase in HDL level were found in the DA group in comparison to the C and CA groups (Table 1).

#### 3.3.5. Blood Glucose Levels

**Figure 6:**
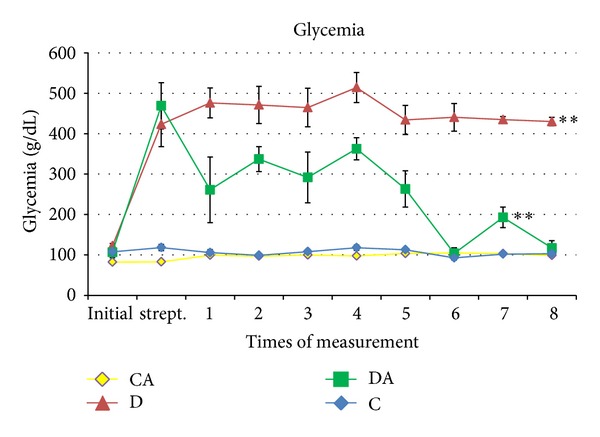
Glicemia in rats submitted to treatments (C: normoglycemic control group having received unaltered commercial chow; CA: normoglycemic control group having received chow with* A. sylvaticus*; D: diabetic group having received unaltered commercial chow; DA: diabetic group having received commercial chow with* A. sylvaticus*). Measurements were taken twice a week for 4 weeks. Values are mean of 5 animals. The first measurement is prior to the initiation of the experiment (time zero). “∗∗” on lines indicates statistically significant differences among all groups (*P* < 0.05; ANOVA + Tukey's test).

One day before the beginning of the experiment, serum glucose concentration was within the normal range (82 to 122 mg/dL). Three days after diabetes induction, increases in glucose concentration were found in the DA and D groups. Glucose was measured twice a week for four weeks. The DA group exhibited a reduction in glycemia in the first week of treatment in comparison to the D group, with continued reduction until the third week. No significant difference was found between the C and CA groups. In contrast, significant differences were found between the C and DA groups (*P* < 0.001) as well as between the D and DA groups (*P* < 0.05) (Figure 6).

### 3.4. Histopathological Analysis

**Figure 7:**
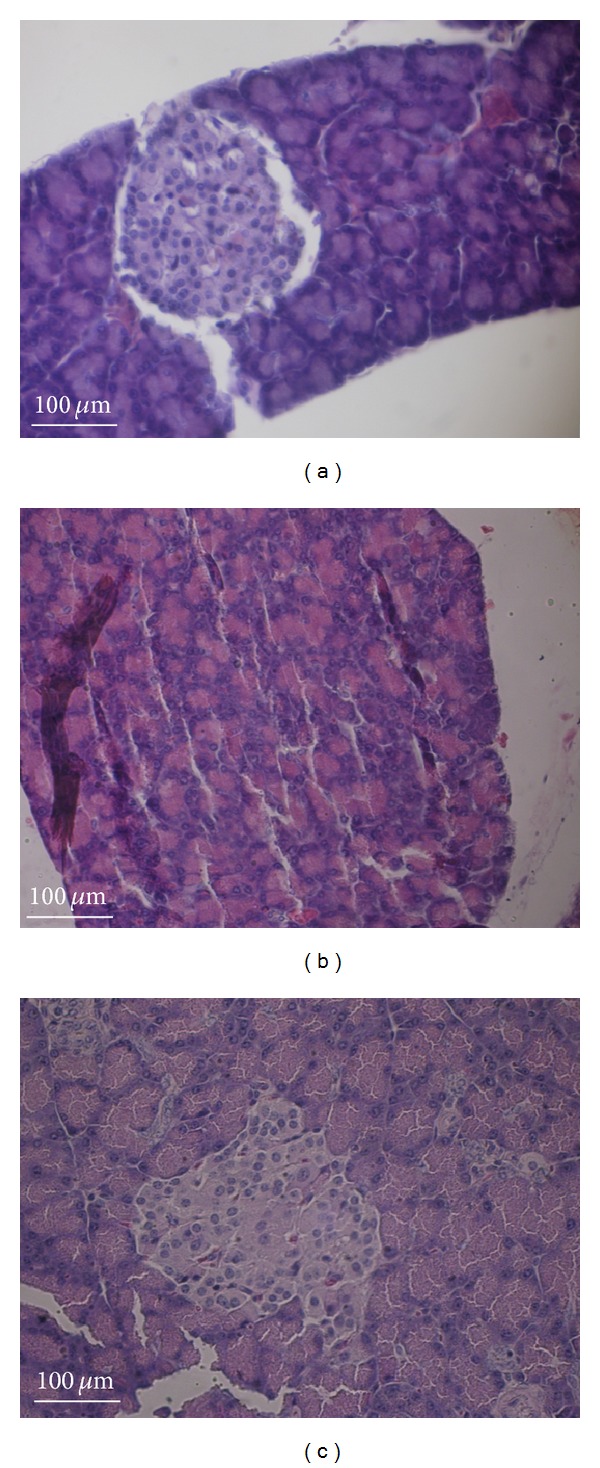
Photomicrographs of pancreas samples; (a) control (C/CA) showing normal round islets of Langerhans; (b) diabetic (D) pancreas showing few *β*-cells and degenerated islets; (c) diabetic animals treated with* A. sylvaticus* (DA) showing hyperplastic pancreatic islets (hematoxylin and eosin, original magnification: 160x).

In both control groups (C and CA), islets of Langerhans were present with normal size and shape. The measurements demonstrated that both control groups had a similar mean area of islets and similar mean number of nuclei (410.5 *μ*m^2^ and 57 nuclei per islet). The samples from the D group exhibited severe degenerative changes in the pancreatic islets, with irregular outlines and decreased nuclei in *β*-cells (mean islet area: 0.62 *μ*m^2^; mean number of nuclei: 20) showing a significant decrease in size in comparison to control group and* Agaricus* group (Kruskal-Wallis test, *P* < 0.05). In contrast, the DA group exhibited nearly normal pancreatic islets slightly oval in shape (mean area: 254.7 *μ*m^2^; mean number of nuclei: 87) (Figure 7 and Table 2).

## 4. Discussion

The present study demonstrates that rats with type 1 diabetes induced by streptozotocin showed elevated levels of cholesterol, HDL, triglycerides, blood glucose, GPT, alkaline phosphatase, and urea and reduced levels of iron and transferrin 30 days following exposure. Dietary supplementation of standard chow with* Agaricus sylvaticus* Schaeffer extracts attenuated these metabolic changes in DM1 animals. Otherwise, normoglycemic animals exhibited no metabolic changes with the use of* A. sylvaticus*. The findings suggest a protective effect of* A. sylvaticus* on the pancreas and in other metabolic biochemical parameters toward homeostasis without change in the nondiabetic individuals that were already in a state of equilibrium (homeostasis).

In the present study, powdered* A. sylvaticus* from the state of Rio Grande do Sul, Brazil, demonstrated complex compounds and proved water soluble. One gram of fungus in 40 mL of water liberated beta-glucan (224.03 mg), proteins (1.15 mg), phenols (0.96 mg), p-coumaric acid, chlorogenic acid, and nonsoluble substances, such as fiber (31 mg) [7].

Many fibers have a polysaccharide *β*-glucan, which is found in a large amount in the cell wall of fungi. Dietary fibers have been strongly implicated in the prevention and treatment of various characteristics of metabolic syndrome. Epidemiologic studies have consistently reported an inverse relationship between dietary fiber and both type 1 and type 2 diabetes mellitus and cardiovascular mortality [19–25]. Babio et al. (2010) examined the effect of different types and sources of dietary fiber on body weight, glucose metabolism, and lipid profile and concluded that the intake of viscous dietary fiber decreases low-density lipoprotein cholesterol and postprandial glucose levels and induces short-term satiety [2].

A number of mechanisms have been suggested to explain the reductions in blood glucose and lipids in the blood as well as body weight through the ingestion of soluble beta-glucan and insoluble fibers. One such mechanism involves the ability of soluble fibers to form viscous solutions. The effects occur in the gastrointestinal tract, with an improvement in laxation and an increase in stool bulk due to the inhibition of the formation of fat in the organism, with metabolic consequences, including improved health [26–28]. In our experiments, while the animals in the C and CA groups had similar increases in weight, the CA group consumed a significantly greater amount of food than the C group, so our results are in accordance with the literature.

The type 1 diabetes model chosen for this study was the use of the chemotherapeutic drug streptozotocin. STZ is selectively toxic to *β*-cells in pancreatic islets and eventually induces an inflammatory process in pancreas producing diabetes in adult rats. STZ-induced hyperglycemia has been described as a good experimental model for the study of DM1 [29].

A possible explanation for the findings with regard to metabolic control by* Agaricus blazei* may reside in the quantity and structure of *β*-glucan in this mushroom. The cell wall *β*-glucans of yeast and fungi consist of 1,3 *β*-linked glucopyranosyl residues, with small numbers of 1,6 *β*-linked branches. These characteristics may influence the immune-modulating effects [12, 19] and can generate an anti-inflammatory response influencing IL-6, prostaglandin D_2_, and leukotriene C_4_ [30].

Moreover, signal mediation is a possible mechanism for the reduction of blood glucose by beta-glucans. Insulin needs to bind to a receptor, which is composed of two extracellular alpha and two transmembrane beta subunits. After the binding of insulin to the extracellular subunit, the intracellular subunit tyrosine kinase is activated [31]. Consequently, four members of the insulin receptor substrate (IRS) family are activated. A phosphorylated reaction from IRS creates sites for binding to another proteins equivalent to phosphatidylinositol 3-kinase (PI3K) with serine/threonine kinase B (Akt). The PI3K/Akt pathway regulates a large number of cell functions, such as apoptosis, cell growth, metabolic effects stimulated by insulin, and the inflammatory process. Decreased PI3K/Akt activity has been shown to play a key role in the pathogenesis of diabetes. Beta-glucans have been demonstrated to increase PI3K/Akt through different receptors [32, 33].

The morphological evaluation of the pancreatic islets revealed compensatory hyperplasia induced by* A. sylvaticus*. This finding suggests a stimulatory effect on these cells. Niwa et al. used the same experimental model in a DM1 study and found similar results with* Agaricus blazei*. The authors suggest that this mushroom has a protective effect on islet cells and increases the secretion of insulin [14]. Thus,* A. blazei* and* A. sylvaticus* lead to a decrease in blood sugar levels in diabetic animals, with intermediate values regarding the area of the islets of Langerhans and number of nuclei between untreated diabetic animals and controls. These results are due to either compensatory hyperplasia of the islets or a protective effect from cell death, which is an important point to investigate in future studies.

The DM1 animals supplemented with* A. sylvaticus* exhibited a significant decrease in total cholesterol, with values approaching those of the control group, as well as a reduction in triglycerides and an increase in HDL. The incorporation of* A. blazei* into food is reported to reduce plasma levels of cholesterol [34] and prevent the development of atheromas [35]. This hypocholesterolemic action may be due to the quantity of fibers and beta-glucans in the mushroom as well as its antioxidant and anti-inflammatory properties [13]. This beneficial effect is important for DM1 patients, as normal blood sugar, normal lipid content, and normal blood pressure are among the goals of medical treatment in such patients.

Water intake was similar in the two control groups (C and CA), but food intake was greater in the CA group. Nonetheless, body weight was maintained in this group. The fact that the CA group ingested more food indicates the palatability was unchanged with the addition of* A. sylvaticus*. Palatability is a main factor that influences food intake by animals [36]. Moreover, the mushroom has a percentage of the soluble fiber beta-glucan, which may help maintain body weight. Depending on the physicochemical properties, fibers have a range of physiological consequences, including viscosity in the upper gastrointestinal tract [37], fermentation in the colon [38], and prebiotic effects [39, 40].

High blood sugar levels can place stress on the kidneys, which can cause serious damage to blood vessels. High concentrations of creatinine and urea constitute markers of kidney dysfunction [41, 42]. In the present study, the animals in the D group exhibited increased urea in comparison to the control groups (C, CA), indicating that diabetes can lead to kidney dysfunction. The significantly lower level of serum urea in the DA group in comparison to the D group suggests that the mushroom exerts an effect on the metabolic control of urea. In contrast, no significant differences among groups were found regarding creatinine levels.

The analysis of transferrin and iron was another important aspect of the present study. Transferrin, which is a glycoprotein, is the main protein in the blood that carries iron throughout the body. Iron is an important element to many functions of the body, but its main role is to be the building block for hemoglobin, which helps red blood cells bind to oxygen for delivery to tissues. Chronic or inflammatory diseases, such as diabetes, result in low transferrin and iron, which is a sign that the liver cells are damaged and the transport of these ions through beta globulin cells in the blood stream is impaired [43, 44]. In the present study, decreased transferrin and iron were found in the animals of the D group, indicating chronic inflammation. However, the incorporation of the mushroom into the food in the DA group led to a statistically significant increase in both transferrin and iron. No significant differences were found between the C and CA groups.

Alkaline phosphatase is distributed in all tissues. An increase in this enzyme in the blood indicates the occurrence of cytolysis and the release of liver cell contents due to damage or necrosis. The increase found in the D group is consistent with results obtained in diabetic patients [45–48]. GPT is found predominantly in the liver at a particular concentration. When liver tissue is damaged and the parenchyma is affected, a greater quantity of this enzyme is released into the blood stream. Thus, the increase in this enzyme found in the D group indicates liver cell damage. However, reductions in both of these enzymes were found in the DA group.

Thinking in all these data together an intraperitoneal injection of the chemotherapy agent streptozotocin damages the pancreas and results in the loss of *β*-cells and degeneration of the islets. This may be through a pathomechanism that involves a local inflammatory response in the pancreas triggered by streptozotocin-induced cell death. These animals are expected to be sick and hence would not grow well despite eating well. The kidneys and liver could also be affected by this insult and hence the increase of urea and liver enzymes and the decrease of transferrin and iron levels. Extracts from* A. sylvaticus* may have a systemic immunomodulatory effect (as opposed to a pancreatic stimulatory effect) that attenuates this inflammatory response, hence resulting in a lesser degree of *β*-cells loss and islet degeneration. The surviving islets and cells would have to compensate and hence become hyperplasic; this is consistent with our findings. It will also lead to less manifestations of diabetic status, as shown in the data, and some degree of kidney and liver protection and hence improved, respectively, urea, enzymes, and transferrin plasma levels.

In conclusion, the present findings demonstrate that* Agaricus sylvaticus* is potentially beneficial in the control of type 1 diabetes by reducing blood glucose, cholesterol, and triglyceride levels, increasing HDL cholesterol and regulating GPT, alkaline phosphatase, iron, transferrin, and urea levels. It may improve the pancreas function increasing the number of the cells in the islets of Langerhans which ameliorate the symptoms of the disease toward the homeostasis.

## Figures and Tables

**Table 1:** Biochemical results of plasma from rats submitted to treatments.

Biochemical tests	C	CA	DA	D	Statistic
Creatinine (mg/dL)	0.30^a^	0.30^a^	0.28^a^	0.30^a^	No diff.
Urea (mg/dL)	38.56^b^	38.38^b^	43.26^b^	81.52^a^	*P* < 0.01
					
Transferrin %	45.00^b^	44.48^b^	47.20^a^	31.00^c^	*P* < 0.01
Iron (mg/dL)	220.00^b^	218.00^b^	241.20^a^	159.66^c^	*P* < 0.01
					
Alkaline phosphatase (mg/dL)	142.00^b^	142.20^b^	166.16^b^	534.80^a^	*P* < 0.01
Glut.-pyr. trans. (GPT mg/dL)	53.33^b^	53.30^b^	57.33^b^	102.80^a^	*P* < 0.01
					
Cholesterol (mg/dL)	60.00^b^	60.50^b^	48.60^c^	85.00^a^	*P* < 0.01
Triglycerides (mg/dL)	65.00^b^	65.30^b^	60.30^c^	86.00^a^	*P* < 0.01
HDL (mg/dL)	27.90^b^	27.80^b^	32.46^a^	19.48^c^	*P* < 0.01

C: normoglycemic control group having received unaltered commercial chow; CA: normoglycemic control group having received chow with *A. sylvaticus*; D: diabetic group having received unaltered commercial chow; DA: diabetic group having received commercial chow with *A. sylvaticus*; values are mean of 5 animals. Different letters on lines indicate statistically significant differences among groups (*P* < 0.01; ANOVA + Tukey's test).

**Table 2:** Area of pancreatic islet and number of nuclei in total area of 110,000 *μ*m^2^.

Experimental groups	Mean islet area (*μ*m^2^)	Maximum diameter (*μ*m^2^)	Minimum diameter (*μ*m^2^)	Mean number of nuclei per islet
C and CA	410.5	1096.7	365.3	57.3*
D	0.62	1.69	0.58	20.0**
DA	254.7	870.3	232.3	87.0*

Legend: C: control; CA: control + *A. sylvaticus*; D: diabetic; DA: diabetic + *A. sylvaticus*. *Statistically nonsignificant, **statistically significant (Kruskal-Wallis test, level of significance = 0.05).

## References

[1] Anderson JW, Davidson MH, Blonde L (2000). Long-term cholesterol-lowering effects of psyllium as an adjunct to diet therapy in the treatment of hypercholesterolemia. *American Journal of Clinical Nutrition*.

[2] Babio N, Balanza R, Basulto J, Bulló M, Salas-Salvadó J (2010). Dietary fibre: influence on body weight, glycemic control and plasma cholesterol profile. *Nutricion Hospitalaria*.

[3] Turner R (1998). Intensive blood-glucose control with sulphonylureas or insulin compared with conventional treatment and risk of complications in patients with type 2 diabetes (UKPDS 33). *The Lancet*.

[4] World Health Organization (2010). *Definition and Diagnosis of Diabetes Mellitus And Intermediate Hyperglycaemia. Report of A WHO/IDF consultation*.

[5] Bach E, Florian M, Wadt N (2013). Vantagens dos cogumelos na alimentação. * Revista Espaço Científico Livre*.

[6] Bach E, Gerenutti M (2010). Biotecnologia aplicada a cogumelos. *Cogumelos Medicinais: Aspectos De Cultivo E Aplicações*.

[7] Bach E, Maureen C, Wadt N (2012). Biotecnologia aplicada em cogumelos: pesquisas realizadas junto a UNINOVE. *Anais VI Simpósio Internacional Sobre Cogumelos No Brasil E V Simpósio Nacional de Cogumelos comestíveis*.

[8] Song HH, Chae HS, Oh SR, Lee HK, Chin YW (2012). Anti-inflammatory and anti-allergic effect of *Agaricus blazei* extract in bone marrow-derived mast cells. *The American Journal of Chinese Medicine*.

[9] Oliveira M, Oliveira E, Lima L, Villas-Boas E (1999). Proximate composition of muschroom (*Agaricus blasei*). *Latin American Symposium on Food Science*.

[10] Firenzuoli F, Gori L, Lombardo G (2008). The medicinal mushroom *Agaricus blazei* murrill: review of literature and pharmaco-toxicological problems. *Evidence-based Complementary and Alternative Medicine*.

[11] Henriques G, Simeon M, Mala A (2008). *In vivo* protein quality of Brazil's mushroom (*Agaricus brasiliensis Wasser* et al.). *Nutrition Journal*.

[12] Hetland G, Johnson E, Lyberg T, Kvalheim G (2011). The mushroom *Agaricus blazei* murill elicits medicinal effects on tumor, infection, allergy, and inflammation through its modulation of innate immunity and amelioration of Th1/Th2 imbalance and inflammation. *Advances in Pharmacological Sciences*.

[13] Novaes MRCG, Novaes LCG, Melo A (2004). Effects of the administration of the *Agaricus sylvaticus* on the hematological and immunological functions of rats with Walker. *Fundamental & Clinical Pharmacology*.

[14] Niwa A, Tajiri T, Higashino H (2011). Ipomoea batatas and *Agarics blazei* ameliorate diabetic disorders with therapeutic antioxidant potential in streptozotocin-induced diabetic rats. *Journal of Clinical Biochemistry and Nutrition*.

[15] Lowry OH, Rosenbrough NJ, Farr AL, Randall RJ (1951). Protein measurement with the Folin phenol reagent. *The Journal of biological chemistry*.

[16] Swain R, Hillis W (1959). The phenolic constituents of *Prunus domestica*. I.—The quantitative analysis of phenolic constituents. *Journal of the Science of Food and Agriculture*.

[17] Lever M (1972). A new reaction for colorimetric determination of carbohydrates. *Analytical Biochemistry*.

[18] Kumar GL, Kiernan JA (2010). *Special Stains and H&E*.

[19] Lu S, Beckles G, Crosson J (2012). Evaluation of risk equations for prediction of short-term coronary heart disease events in patients with long-standing type 2 diabetes: the Translating Research into Action for Diabetes (TRIAD) study. *BMC Endocrine Disorders*.

[20] Maiti R, Das UK, Ghosh D (2005). Attenuation of hyperglycemia and hyperlipidemia in streptozotocin-induced diabetic rats by aqueous extract of seed of *Tamarindus indica*. *Biological and Pharmaceutical Bulletin*.

[21] Marlett J, Cho SS, Dreher ML (2011). Dietary fiber and cardiovascular disease. *Handbook of Dietary Fiber*.

[22] Mizuno M, Nishitani Y (2013). Immunomodulating compounds in Basidiomycetes. *Journal of Clinical Biochemistry and Nutrition*.

[23] Murali B, Goyal RK (2001). Improvement in insulin sensitivity by losartan in non-insulin-dependent diabetic (NIDDM) rats. *Pharmacological Research*.

[24] Nirmala, Saroja S, Vasanthi H, Lalitha G (2009). Hypoglycemic effect of Basella rubra in streptozotocin—induced diabetic albino rats. *Journal of Pharmacognosy and Phytotherapy*.

[25] Wong ND, Patao C, Malik S, Iloeje U (2014). Preventable coronary heart disease events from control of cardiovascular risk factors in US adults with diabetes (Projections from Utilizing the UKPDS Risk Engine). *American Journal of Cardiology*.

[26] Brown L, Rosner B, Willett WW, Sacks FM (1999). Cholesterol-lowering effects of dietary fiber: a meta-analysis. *American Journal of Clinical Nutrition*.

[27] Roberfroid MB (2007). Inulin-type fructans: functional food ingredients. *Journal of Nutrition*.

[28] Butt MS, Tahir-Nadeem M, Khan MKI, Shabir R, Butt MS (2008). Oat: unique among the cereals. *European Journal of Nutrition*.

[29] Punithavathi VR, Prince PSM, Kumar R, Selvakumari J (2011). Antihyperglycaemic, antilipid peroxidative and antioxidant effects of gallic acid on streptozotocin induced diabetic Wistar rats. *European Journal of Pharmacology*.

[30] Chen J, Seviour R (2007). Medicinal importance of fungal *β*-(1 → 3)
, (1 → 6)-glucans. *Mycological Research*.

[31] Ouedraogo M, Baudoux T, Stévigny C (2012). Review of current and “omics” methods for assessing the toxicity (genotoxicity, teratogenicity and nephrotoxicity) of herbal medicines and mushrooms. *Journal of Ethnopharmacology*.

[32] Hsu M-J, Lee S-S, Lin W-W (2002). Polysaccharide purified from Ganoderma lucidum inhibits spontaneous and Fas-mediated apoptosis in human neutrophils through activation of the phosphatidylinositol 3 kinase/Akt signaling pathway. *Journal of Leukocyte Biology*.

[33] Önning G, Wallmark A, Persson M, Åkesson B, Elmståhl S, Öste R (1999). Consumption of oat milk for 5 weeks lowers serum cholesterol and LDL cholesterol in free-living men with moderate hypercholesterolemia. *Annals of Nutrition and Metabolism*.

[34] Kweon M-H, Kwon S-T, Kwon S-H, Ma M-S, Park YI (2002). Lowering effects in plasma cholesterol and body weight by mycelial extracts of two mushrooms: *Agaricus blazai* and *Lentinus edodes*. *Korean Journal of Microbiology and Biotechnology*.

[35] Sahyoun NR, Jacques PF, Zhang XL, Juan W, McKeown NM (2006). Whole-grain intake is inversely associated with the metabolic syndrome and mortality in older adults. *American Journal of Clinical Nutrition*.

[36] Percario S, Odorizzi VF, Souza DR (2008). Edible mushroom Agaricus sylvaticus can prevent the onset of atheroma plaques in hipercholesterolemic rabbits. *Cellular and Molecular Biology*.

[37] Darwiche G, Björgell O, Almér L-O (2003). The addition of locust bean gum but not water delayed the gastric emptying rate of a nutrient semisolid meal in healthy subjects. *BMC Gastroenterology*.

[38] Wong JMW, De Souza R, Kendall CWC, Emam A, Jenkins DJA (2006). Colonic health: fermentation and short chain fatty acids. *Journal of Clinical Gastroenterology*.

[39] Slavin JL (2005). Dietary fiber and body weight. *Nutrition*.

[40] Volman JJ, Ramakers JD, Plat J (2008). Dietary modulation of immune function by *β*-glucans. *Physiology and Behavior*.

[41] Hi EMB, Bach EE, Ogata TS, Gerenutti M (2010). Avaliação do efeito protetor de extratos do cogumelo *Agaricus sylvaticus* em ratos inoculados com agente carcinogênico (Pristane). *Cogumelos Medicinais: Aspectos de Cultivo e Aplicações*.

[42] Sherlock S (1985). *Diseases of the Liver Biliary Tract and Pancreas*.

[43] Swaminathan S, Fonseca VA, Alam MG, Shah SV (2007). The role of iron in diabetes and its complications. *Diabetes Care*.

[44] Zhao Z, Li S, Liu G (2012). Body iron stores and heme-iron intake in relation to risk of type 2 diabetes: a systematic review and meta-analysis. *PLoS ONE*.

[45] Falchuk KR, Conlin D (1993). The intestinal and liver complications of diabetes mellitus. *Advances in Internal Medicine*.

[46] Sando H, Lee Y, Twamoto Y, lkeuchi M, Rosaka K (1980). lsopotereno stimulated C-peptide and insulin secretion in diabetic and normal subjects. Decreased Hepatic excretion of endogenous insulin in Diabetes. *The Journal of Clinical Endocrinology & Metabolism*.

[47] Shinde UA, Goyal RK (2003). Effect of chromium picolinate on histopathological alterations in STZ and neonatal STZ diabetic rats. *Journal of Cellular and Molecular Medicine*.

[48] Czech MP, Corvera S (1999). Signaling mechanisms that regulate glucose transport. *The Journal of Biological Chemistry*.

